# Supplementary figures and images for: The Preparation and Biological Testing of Novel Wound Dressings with an Encapsulated Antibacterial and Antioxidant Substance
Source: Nanomaterials (Basel). 2022 Oct 29;12(21):3824. doi: 10.3390/nano12213824 (PMC9656126; doi:10.3390/nano12213824)

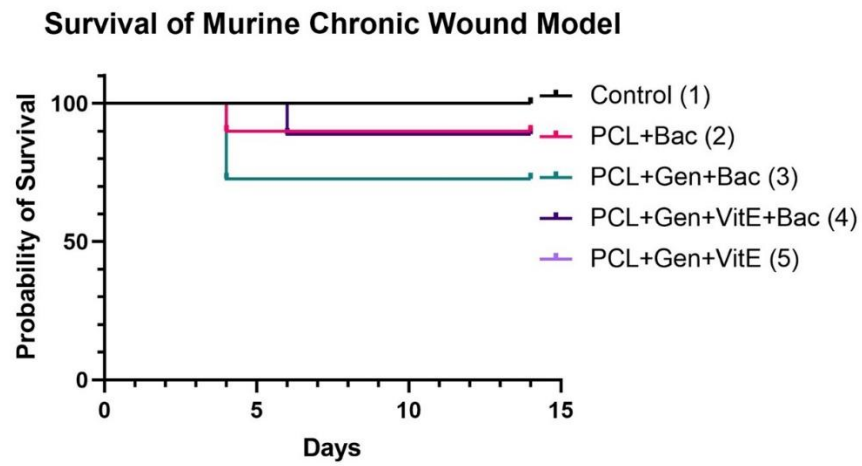

**Figure S1.** Survival of murine chronic model in a 14-day in vivo experiment (% alive animals).

Supplement: Supplementary file 1 [file nanomaterials-12-03824-s001.zip › nanomaterials-1991016-supplementary.pdf]
